# Optimal subscription models to pay for antibiotics

**DOI:** 10.1016/j.socscimed.2022.114818

**Published:** 2022-04

**Authors:** Euan Barlow, Alec Morton, Itamar Megiddo, Abigail Colson

**Affiliations:** Department of Management Science, Strathclyde Business School, University of Strathclyde, Glasgow, UK

**Keywords:** Subscription payment models, use of novel antibiotics, antimicrobial resistance, evidence-based health policies

## Abstract

Novel subscription payment schemes are one of the approaches being explored to tackle the threat of antimicrobial resistance. Under these schemes, some or all of the payment is made via a fixed “subscription” payment, which provides a funder unlimited access to the treatment for a specific duration, rather than relying purely on a price per pill. Subscription-based schemes guarantee pharmaceutical firms income that incentivises investment in developing new antibiotics, and can promote responsible stewardship. From the pharmaceutical perspective, revenue is disassociated from sales, removing benefits from push marketing strategies. We investigate this from the funder perspective, and consider that the funder plays a key role in promoting responsible antibiotic stewardship by choosing the price per pill for providers such that this encourages appropriate antibiotic use. This choice determines the payment structure, and we investigate the impact of this choice through the lens of social welfare. We present a mathematical model of subscription payment schemes, explicitly featuring fixed and volume-based payment components for a given treatment price. Total welfare returned at a societal level is then estimated (incorporating financial costs and monetised benefits). We consider a practical application of the model to development of novel antibiotic treatment for Gonorrhoea, and examine the optimal treatment price under different parameterisations. Specifically, we analyse two contrasting scenarios - one where a new antibiotic's prioritised role is reducing transmission, and one where a more pressing requirement is conserving the antibiotic as an effective last defence. Critically, this analysis demonstrates that effective roll-out of a subscription payment scheme for a new antibiotic requires a comprehensive assessment of the benefits gained from treatment. We discuss the insights this work presents on the nature of these payment schemes, and how these insights can enable decision-makers to take the first steps in determining effective structuring of subscription payment schemes.

## Highlights

• Policymakers and insurers are currently designing antibiotics subscription schemes.
• Social welfare returned from these payment schemes is modelled mathematically.
• Payment structures maximising social welfare are identified under different scenarios.
• If reduced transmission is a priority, subscription-only payments are near-optimal.
• If preserving an antibiotic is a priority, optimal structures include per-use payments.

## 1 Introduction

Growing concern about a shortage of effective antibiotics due to the increase in resistance rates has led policy makers to seek policy instruments which encourage pharmaceutical companies to expedite (and in some cases re-initiate) their antibiotic discovery programmes. Antibiotics have been a pillar in modern medicine, but their effectiveness erodes with use. In large part this erosion stems from a market failure, where individual behaviour is contrary to the common good. The use of an antibiotic treats the antibiotic-susceptible strains of bacteria, and natural selection then promotes the growth of the remaining, resistant, strains. Provider and patient incentives for positive outcomes for the given patient neglect this wider impact of consumption. Pharmaceutical companies are incentivised to increase their revenues, through either increasing sales or increasing prices. These incentives lead to the tragedy of the commons, likened to climate change, and with common overuse and misuse (Roope et al., 2019). In this analogy, an effective antibiotic treatment is the scarce resource (the commons), and overuse and misuse of this resource leads to depletion (through the rise of antimicrobial resistance).

In parallel, a shrinking pipeline of novel antibiotics is failing to keep pace with the emergence and spread of resistant bacteria (Årdal et al., 2018). Fast paced discovery and development of antibiotics spanned from the 1940s until the 1980s, when increasing regulatory costs, a belief that sufficient antibiotics had been developed, competition with existing antibiotics, and a host of other factors slowed down innovation (Årdal et al., 2020). Today, the revenues from antibiotics compare poorly to other drug classes, and they do not cover the costs to develop, bring to market, and deliver an antibiotic (Towse et al., 2017; Outterson and Rex, 2020). A patent-based business model, which provides exclusivity and increases revenues through either more sales or a higher price, works well for many other therapeutics. Uniquely, however, novel antibiotics are used sparingly in the first years after coming to market to preserve their effectiveness, and their sales volume is therefore low. Traditional funding mechanisms ignore resistance and preservation of effectiveness, treating them as externalities - there is no compensation for these low sales to recognise the innovation that yields the effective novel antibiotic treatment (Outterson and Rex, 2020). Furthermore, the value of this effectiveness is not currently recognised through prices, and demonstrating continued effectiveness (or a lack of resistance) would first require population studies. Combining these factors with trial design limitations, incentives of hospital reimbursement structures, and expectations that infections are cured cheaply, leads to antibiotic prices being typically low (Årdal et al., 2020; Outterson and Rex, 2020). The result is that the current pipeline of antibiotics in development is insufficient to address projected clinical needs (Renwick et al., 2016; Simpkin et al., 2017).

Recent projects (Årdal et al., 2018; Rothery et al., 2018) explicitly define values associated with antibiotics that are typically ignored, and arise due to the tension between the private (patients) and societal (the wider impact of consumption) perspectives. Firstly, novel, effective antibiotics prevent “transmission” and thus limit the spread of disease. They increase the “diversity” of our existing portfolio of drugs, increasing the ability to treat infections resistant to this portfolio while alleviating the selection pressure on other drugs. They “enable” procedures that otherwise may be deemed too risky. Finally, they provide “insurance” that effective treatment can be accessed in a future with uncertain resistance levels to our existing portfolio.

Researchers have proposed push and pull mechanisms to incentivise development of novel antibiotics, that reflect the societal value of these products without overpaying for them (Årdal et al., 2020). Push mechanisms provide direct or indirect upfront savings to reduce the costs of research and development and the barriers to entry, especially for SMEs (Luepke et al., 2017; Renwick et al., 2016; Roope et al., 2019). Pull mechanisms reward successful products by increasing returns and/or reducing the uncertainty of returns on investment (Luepke et al., 2017; Renwick et al., 2016; Roope et al., 2019; Årdal et al., 2020). Though both push and pull mechanisms are important, for a variety of reasons push incentives have been more widely implemented (Simpkin et al., 2017; Årdal et al., 2020). The lack of pull incentives has resulted in the bankruptcy filing of smaller companies such as Achaogen, which brought a novel drug to market but only had revenues of $800,000 in the first year (Outterson and Rex, 2020).

Of the various pull mechanisms that have been proposed, subscription style business models as proposed by Towse et al. (2017) are currently being piloted in the UK and Sweden (Department of Health and Social Care, GOV.UK, 2019; Public Health Agency of Sweden, 2019). These subscription payment models consider that the total payment to manufacturers of a novel antibiotic can be decomposed into a subscription component (a fixed lump-sum) and a variable component (dependent on the volume of treatment allocations). The financial gains for a pharmaceutical company are therefore at least partially delinked (separated) from the volume of sales of the antibiotic, and these models have therefore been referred to as delinked models (Outterson et al., 2016). The delinked models provide society access to effective antibiotics (Towse et al., 2017) and remove pharmaceutical incentives to increase sales volumes, while ensuring that successful products can generate revenue from subscription payments. However, appropriate antibiotic use still needs to be encouraged.

The responsibility to encourage appropriate use could be put on both pharmaceuticals and providers. For example, tying the value of a contract to ongoing drug effectiveness to incentivise companies to conserve effectiveness (Morel et al., 2020), or taxing providers to incentivise reduced consumption (Roope et al., 2019). Effectively deploying these schemes to control appropriate use could be challenging, however, and the alternative considered here places the core burden of promoting appropriate use directly on funders and policy makers, such as national governments. These are best positioned to control usage through targeted treatment pricing, and are arguably the direct beneficiaries of sustained and effective antibiotic treatment options.

A key challenge to supporting funders in implementing such subscription models is selection of the modelling strategy used to frame policy decisions and establish their effectiveness. Of the different types of societal value that are associated with antibiotic usage, the impact of resistance has received most attention in the literature. Coast et al. (1996) provide one of the earliest economic considerations of antimicrobial resistance, making the argument for incorporating resistance into economic evaluations of antibiotics. In Coast et al. (1998) the same authors go on to explicitly feature resistance as a negative externality in a cost-benefit framework for antimicrobial usage. They reflect on the impact of this externality, and on the effectiveness of different types of economic policy at reducing resistance. Coast et al. (2002) further consider economic assessment of health policy interventions targeted at reducing antibiotic resistance. They specifically compare interventions targeted at avoiding either resistance emergence or resistance transmission. Throughout this body of work (Coast et al., 1996, 1998, 2002), the examinations presented by the authors represent some of the earliest steps to establish a structured assessment of the economic impact of antibiotic resistance, to ultimately enable this impact to be incorporated into health policy decision making.

Gersovitz and Hammer (2003) present an early examination on the contrast between individual behaviour and public policy, in relation to choices that impact the therapy for and prevention of infectious diseases. They discuss that in the presence of externalities these two perspectives may not be aligned, and governments would seek policy interventions to align individual behaviour with outcomes that are targeted at greater societal value. To identify and evaluate these, Gersovitz and Hammer (2003) promote the role of models combining economic perspectives at the individual level with epidemiological models of transmission dynamics at the societal level. This framing directly applies to pricing for a novel antibiotic, and the use of policy to attain outcomes that target increased societal value. Numerous studies have used this combined modelling approach to conduct an economic evaluation of policy decisions. Examples of this approach include Herrmann (2010); Herrmann and Nkuiya (2017); Chehrazi et al. (2019) (see also references therein). In each case, the authors present a combined economic-epidemiological model to consider specific policy decisions, and to explore the effect of resistance on the policy effectiveness. These models of transmission dynamics are complex and may be difficult to parameterise accurately in practice, yet still do not comprehensively represent all aspects of the societal values discussed above that are gained from effective antibiotic treatments.

In a more general health context, Barros (2011) presents a model of a risk-sharing arrangement between a health care provider and a treatment manufacturer. The probability of gaining a fixed treatment benefit varies across the infected population, and the analysis is framed around a treatment price variable which drives the treatment policy. Various extensions to this modelling are presented in Mahjoub et al. (2018) and Critchley and Zaric (2019).

In this paper, we present a payment model to interrogate the optimality of a subscription-based payment schedule, from a social welfare perspective. Our approach set out in Section 2 formalises the model proposed in Towse et al. (2017), and is novel in that we consider the problem from an economic perspective, which abstracts from the complex infectious disease transmission modelling that features in other comparable works. Our modelling approach is inspired by the approach of Barros (2011) to maximise the social planner's welfare when the treatment outcome is uncertain. We use a similar general framing, but tailor this to a subscription-based payment context, and consider the effect of treatment pricing on social welfare in terms of returned private and societal values.

## 2 Model for subscription payment and selective treatment

### 2.1 Modelling approach

We consider that a specific novel antibiotic ABX has passed medicines regulation and is licensed for human use. We consider a target population comprising those individuals who are potential candidates to receive treatment ABX (based on the properties of ABX and the expected infection). We assume that the medical practitioner will choose to treat an individual from the candidate population with ABX if the case is sufficiently severe - or more precisely, if the benefits to the individual from treatment are sufficiently high. Otherwise, the individual will be treated with an alternative treatment option.

From the perspective of a health policy decision-maker, we assume that the key mechanism in controlling the extent to which ABX is used for treatment is the treatment price of ABX. This assumption resonates with the view expressed in Rex and Outterson (2020) that, for the UK pilot scheme, the NHS aim to set a unit price for the new antibiotic that “encourages appropriate use while discouraging inappropriate use”. We recognise that in the UK system individual clinicians would not make a decision based on price. Nevertheless, pricing can help send a message about the intensity of the need to conserve ABX, and that message will shape individual clinicians indirectly through institutional incentives.

Our model measures the effectiveness of a policy choice in setting the treatment price of ABX. This choice controls both the subscription-based payment schedule for ABX (in terms of the lumpsum and volume-based components) and the stewardship of ABX (by setting the minimum health benefits expected to be returned from treatment). Similar to the approach of Towse et al. (2017), we assume that the payment to be made to the manufacturer is fixed and represents an assessment of the total payment that is necessary to compensate the manufacturer's innovation costs and supply a reasonable margin. This is additionally aligned with the structure of the UK pilot scheme, which has fixed the total annual payment to a manufacturer. Within this constraint, our model assesses the effectiveness of a policy choice in terms of the difference between the total monetised health benefits returned from individuals treated with ABX and the manufacturer payment. As we measure the health benefits returned from treatment at a societal level, we define this assessment as the social welfare returned from the policy choice. We recognise, however, our assumption that manufacturer costs are compensated (plus a reasonable margin) does not measure the welfare of the pharmaceutical firm. A more comprehensive social welfare assessment could capture this explicitly.

For the UK pilot scheme, the maximum total annual payment to a manufacturer has been pre-determined. The payment actually made to a manufacturer will be informed by an assessment of the value of health benefits provided by a new antimicrobial, and will capture both private and societal value components. This annual payment will be fixed for an initial three-year contract. At that point the government may extend the contract up to a period of 10 years (National Institute for Health and Care Excellence, 2020), with the decision to extend presumably partially informed by a cost-effectiveness assessment of the initial contract. Our model's intended use is to support payment scheduling decisions during this initial three-year contract, or during a subsequent extension once the manufacturer payment is re-evaluated. In either case, with the manufacturer payment decided, the role of our model would be to extract the maximum social welfare from usage of the new antibiotic. Our model is not intended to assess the cost-effectiveness of the total annual manufacturer payment, with detailed measurement of the constituent costs and benefits. Rather, our model takes the manufacturer payment and the societal benefit gained from antibiotic use as inputs, and then calculates the maximum social welfare that can be returned in this context through optimal choice of the treatment price. Our model is therefore framed to identify whether the total payment to the manufacturer is greater or less than the health benefits that are returned (see equation (1)). We intend to address this question further in future work by developing a formal structure of the societal value measurement. This would provide a more appropriate assessment of the value of the new antibiotic than is currently standard. Rex and Outterson (2020) speculate that the unit price set by the NHS to encourage only appropriate use could be a small percentage above the price of an alternative generic antibiotic. This should be sufficiently high that the new antibiotic would only be used when it is appropriate to do so, but still cheap enough that it can be used when needed. Our model framing is consistent with this view and could be used to inform the approach to setting an appropriate level of usage for the new drug. While our model is positioned to inform this unit pricing decision (with regard to maximising social welfare), various other factors would likely also influence the decision. A full consideration of the policy-maker's decision process, however, is beyond the scope of this work.

### 2.2 Model notation and setup

We consider that the target population, *P*, is of size *N*, and define the treatment price as *T*. Our model features two separate functions that are used to evaluate the treatment benefits for an individual - one which measures the value of the treatment that is received (from the patient perspective), and one which informs the decision to allocate treatment (from the prescriber perspective). We then investigate the nature of the optimal treatment price and optimal returned social welfare, under different features of these valuation functions. We define *T* as the incremental treatment price of ABX, as compared to the price of the alternative treatment option, and assume that the prices for each drug include the costs of production, distribution, storage and administration to patients. We assume that the benefits (from both patient and prescriber perspectives) are the incremental benefits, as compared to the corresponding benefits of the alternative treatment option.

We follow the convention that *X*_*.*_ represents a random variable, *F*_*.*_ its cumulative distribution such that $F_{.}:R:\to \left[0,1\right]$ and, unless otherwise stated, *f*_*.*_ its density function such that $f_{.}\left(x\right):R:\to \left[0,1\right]$. This target population can be decomposed into two sub-populations - those allocated treatment and those refused treatment, denoted as **P**^*A*^ and **P**^*R*^, respectively. The social planner's welfare function is a measure of the total financial outlay to the manufacturer of ABX, and the monetised health benefits returned from the treated cases of **P**^*A*^. We write this as

(1) $$W=-C_{tot}+B,$$

for monetised health benefits *B*. In keeping with the approach of Towse et al. (2017), we suppose that an assessment *C*_*tot*_ has been made of the total payment which is necessary to compensate the manufacturer's innovation costs and supply a reasonable margin. This will comprise two separate payments

(2) $$C_{tot}\left(T,C_{L},P^{A}\right)=C_{L}+T|P^{A}|,$$

where *C*_*L*_ represents the lumpsum payment, |.| denotes the cardinality of a set, and the second term therefore represents the volume-based payment calculation: the number of cases receiving treatment at a cost of *T*. We assume that the health system applies a cost-benefit test when determining eligibility for treatment, with treatment only allocated in those cases where the benefit value is returned greater than the treatment price *T*. The outcome of this allocation test is described as a random variable *X*_*A*_ where density function *f*_*A*_(*x*) models the percentage of the target population which returns *X*_*A*_ = *x* from the cost-benefit test. The treated population is thus defined as **P**^*A*^(*T*) = {**P**|*X*_*A*_ ≥ *T*}, and the percentage of the population allocated treatment is calculated as

(3) $${\bar{F}}_{A}\left(T\right)=\int_{T}^{\infty }f_{A}\left(x\right)\;dx,$$

where ${\bar{F}}_{A}\left(x\right)=1-F_{A}\left(x\right)$ denotes the complimentary cumulative (or exceedence) distribution. The volume-based payment can now be expressed as $TN{\bar{F}}_{A}\left(T\right)$. Under the assumption that *C*_*tot*_ is fixed, *T* becomes the sole decision variable, and the lumpsum payment is adjusted to balance equation (2) as follows,

(4) $$C_{L}\left(T,C_{tot}\right)=C_{tot}-TN{\bar{F}}_{A}\left(T\right).$$

The monetised health benefit *B* is described similarly to the treatment allocation test; we model this as a random variable *X*_*B*_ where density function *f*_*B*_(*x*) models the percentage of the target population which receives benefit *X*_*B*_ = *x* from the treatment. The total monetised health benefit returned from the treated population can be defined as

(5) $$B\left(T\right)=N\int_{P^{A}\left(T\right)}xf_{B}\left(x\right)\;dx$$

Substituting equation (5), (1), the social planner's welfare function *W* is defined as

(6) $$W\left(T\right)=-C_{tot}+N\int_{P^{A}\left(T\right)}xf_{B}\left(x\right)\;dx.$$

From a welfare economics or cost-benefit perspective, the decision to introduce payment scheme {*T*, *C*_*L*_} should be guided by whether *W* > 0. Where possible an optimal payment scheme maximising *W* is desirable, namely identifying

(7) $$T^{*}=\arg\;\max_{T}W\left(T\right),$$

such that T* is the optimal treatment price.

In the following section we explore the optimality of the payment scheme under different formulations of the value functions that are used to determine treatment allocations decisions, and to determine the realised monetary benefit from a treatment allocation. Specifically, two alternative valuation functions are explored - a societal value function and a private value function, as described in Section 1. We describe these values per case of the target population as the random variables *X*_*S*_ and *X*_*P*_ for the societal and private values, respectively. The societal value has density function *f*_*S*_(*x*) which models the percentage of the target population receiving societal value *X*_*S*_ = *x* from the treatment. Similarly, the private value has density function *f*_*P*_ which models the percentage of the target population receiving private value *X*_*P*_ = *x* from the treatment, and we note that $f_{P}\left(x\right):R^{+}:\to \left[0,1\right]$. Additionally, we introduce the mapping function *X*_*S*_ = *φ*(*X*_*P*_); if it exists, $\varphi :R^{+}:\to R$ defines the societal value for a case of the target population, based on the corresponding private value. Societal values are considered to be “fully loaded”: these may be negative if the broader consequences of use for less severe patients are outweighed by the damage caused by the spread of resistance. In contrast, the private values are not “fully loaded” and take into account only the benefits which accrue to the individual patient; the individual hospitals or even clinicians who make the eligibility decision regard the broader social benefits as externalities.

## 3 Optimal payment scheme

We consider the case where a societal perspective is used to evaluate the monetised health benefit returned from a treatment allocation according to a societal value function, as defined in Section 2 with outcome *X*_*S*_. To clarify terminology, we restrict “societal” to reference this value function, and use “social” to reference the welfare. With this modification the social planner's welfare is, from equation (6)

(8) $$W\left(T\right)=-C_{tot}+N\int_{P^{A}\left(T\right)}xf_{S}\left(x\right)\;dx.$$

In the remainder of this section, we consider equation (8) under various assumptions in order to explore two key practical features which we anticipate could arise when this approach is applied in practice:

● That the value function used to evaluate whether treatment should be allocated is different to the value function used to evaluate the monetised health benefit returned from treatment
● That there may be some level of stochastic dependence between these value functions

In Section 3.1 we first assume that these two value functions are equivalent, and identify some basic properties of the optimal treatment strategy. Section 3.2 relaxes this assumption, and considers two distinct value functions. The impact on the optimal treatment strategy is demonstrated, and general properties of this formulation are discussed. Sections 1, 2, 3 then consider the impact of additional modelling assumptions on the form of, and relationship between, the two value functions. In Section 3.2.1 the two value functions are assumed to be stochastically dependent. This enables a closed-form solution to be identified for the optimal treatment strategy under a given value function. Section 3.2.2 extends this by further assuming that both value functions are normally distributed, and derives simple analytic expressions for the optimal treatment strategy. Finally, Section 3.2.3 retains the assumption of normality but relaxes the assumption of dependence between the value functions. A simulation model is used to explore the impact on the optimal treatment strategy as this dependence is varied. This final model provides the greatest flexibility and applicability in a practical context, and interpretation of the simulation outputs (as presented in Section 4.3.2) utilises the analytical insights gained throughout the preceding model variants.

### 3.1 Optimal policy with treatment decisions from a societal perspective

We first consider the simple case where treatment allocation decisions are also based on the societal value. The outcome of the treatment allocation test outlined in Section 2 is therefore defined with realisation *X*_*A*_ = *X*_*S*_, density function *f*_*A*_ = *f*_*S*_, and cumulative distribution *F*_*A*_ = *F*_*S*_. Equations (3), (4) are updated accordingly. In this case the treated population can be written directly in terms of the range of *X*_*S*_ as, **P**^*A*^(*T*) = {**P**|*X*_*S*_ ≥ *T*}. The integral limits for the social planner's welfare function (8) therefore simplify, giving

(9) $$W\left(T\right)=-C_{tot}+N\int_{T}^{\infty }xf_{S}\left(x\right)\;dx.$$

Taking derivatives and first order conditions of (9) yields

(10) $$\frac{dW}{dT}=-NTf_{S}\left(T\right).$$

Therefore:

Proposition 1 *With a social welfareW*(*T*) *defined by equation* (9)*, the optimal treatment price is* T* = 0*. An optimal pricing scheme is therefore* *C*_*tot*_ = *C*_*L*_ *- that is, the payment to ABX manufacturer is entirely comprised of the lumpsum (subscription) component, and has no volume-based contribution.*

Proof *See*Appendix A. *■*

To implement such a payment scheme, every member of the target population returning a non-negative societal value from treatment would be allocated the treatment. This optimum is expected from basic economic theory - any higher price would be a monopolistic restriction on value-adding consumption and so would destroy value.

### 3.2 Optimal policy with treatment decisions from a private perspective

We now extend the scenario outlined in Section 3.1, by considering an alternative value function to determine treatment allocation decisions. Specifically, it may be that when the health system applies its cost-benefit test in determining eligibility for treatment allocation it uses an individual level private value function, as defined in Section 2 with outcome *X*_*P*_. The outcome of the treatment allocation test outlined in Section 2 is therefore defined with realisation *X*_*A*_ = *X*_*P*_, density function *f*_*A*_ = *f*_*P*_, and cumulative distribution *F*_*A*_ = *F*_*P*_. The treated population is thus defined as **P**^*A*^(*T*) = {**P**|*X*_*P*_ ≥ *T*}. Equation (3) is now updated as

(11) $${\bar{F}}_{P}\left(T\right)=\int_{T}^{\infty }f_{P}\left(x\right)\;dx,$$

giving a volume-based payment of

(12) $$C_{V}\left(T\right)=TN{\bar{F}}_{P}\left(T\right),$$

and from equation (4) the lumpsum payment is

(13) $$C_{L}\left(T,C_{tot}\right)=C_{tot}-TN{\bar{F}}_{P}\left(T\right).$$

Applying the intuition for Proposition 1 to equation (8), with fixed *C*_*tot*_ and non-negative density function for the societal value, maximisation of welfare *W* again coincides with integrating over the range of positive *X*_*S*_ values. That is

(14) $$W^{*}=-C_{tot}+N\int_{0}^{\infty }xf_{S}\left(x\right)\;dx.$$

In this setting, however, different criteria are used to make allocation decisions and to measure returned benefits. Therefore setting a threshold for the returned benefits (at *X*_*S*_ = 0) does not necessarily translate into a threshold level for allocation decisions (that is, in terms of a required value of *X*_*P*_). Integrating over the entire region of the integral in equation (14) is therefore not necessarily attainable. Additional information on the societal value returned from treated individuals is therefore required to make further analytical progress towards optimising the social planner's welfare. In sections 1, 2, 3, we present formulations of the welfare function (8) under different specifications of the relationship between the societal and private value functions.

#### 3.2.1 Optimality with stochastic dependence between societal and private values

We consider the case where there is a one-to-one mapping from the private value to the societal value, such that societal value for individuals is a monotonically increasing function of the corresponding private value. That is, the ranking of individuals is unchanged between societal and private values. We express this mathematically as the assumption that societal value and private value share a perfect rank correlation across the target population. It is apparent that this assumption of perfect rank correlation is in fact a sufficient condition for a treatment allocation decision thresholded at some value of *T* to return the maximum social planner's welfare, as given in equation (14).

We operationalise the perfect rank correlation assumption by specifying the mapping function *φ* as

(15) $$\varphi \left(X_{P}\right)={\bar{F}}_{S}^{-1}{\bar{F}}_{P}\left(X_{P}\right),$$

and the societal value generated by treating the marginal individual is *φ*(*T*). The social planner's welfare function is therefore defined from equation (8) as

(16) $$W\left(T\right)=-C_{tot}+N\int_{\varphi \left(T\right)}^{\infty }xf_{S}\left(x\right)\;dx.$$

With a fixed payment to the manufacturer, *C*_*tot*_, and recognising that the integrand is independent of *T*, application of the Leibniz integral rule to equation (16) produces

(17) $$\frac{\partial }{\partial T}W\left(T\right)=-N\varphi \left(T\right)f_{S}\left(\varphi \left(T\right)\right)\frac{d\varphi \left(T\right)}{dT}.$$

We can expect the following results:

Proposition 2

1. *If there is perfect rank correlation between an individual’s societal and private values*, *X*_*S*_*andX*_*P*_, *respectively*, then the total social welfare is maximised forT* *such thatφ*(T*) = 0. *That is*, $T^{*}={\bar{F}}_{P}^{-1}{\bar{F}}_{S}\left(0\right)$.
2. *If the rank correlation betweenX*_*S*_*andX*_*P*_*is imperfect*, *then the maximum social welfare as defined in* equation (14) *may be unattainable*.
3. *IfX*_*P*_ (*X*_*S*_) *stochastically dominates* *X*_*S*_ (*X*_*P*_), *then* *φ*(*T*) ≤ (≥)*T*.
4. *Iff*_*P*_(*x*) = *f*_*S*_ (*x* − *c*) *for* c∈R, *then* *φ*(*T*) = *T* − *c*.

Proof *See*Appendix A. *■*

Exploiting standard definitions for the expected value of a random variable with truncated distribution, equation (14) therefore results in the maximum welfare

(18) $$W^{*}=-C_{tot}+N\left(1-F_{s}\left(0\right)\right)E\left[X_{S}|X_{S}>0\right].$$

This alternative formulation provides a useful mechanism for determining W* in cases where the expectation for a truncated societal value distribution are well-defined. It is evident from equations (17), (18) that the societal, rather than private, value distribution is the key factor influencing the maximised social welfare.

#### 3.2.2 Optimality with defined societal and private value distributions

In order to make analytical progress, we next consider the case where the societal and private values are normally distributed as $X_{S}∼N\left(\mu_{S},\sigma_{S}\right)$ and $X_{P}∼N\left(\mu_{P},\sigma_{P}\right)$. Note that we retain the assumption that the societal and private values have perfect rank correlation. As shown in Appendix B, the optimal treatment price that satisfies *φ*(T*) = 0 is found to be

(19) $$T^{*}=\mu_{P}-\frac{\sigma_{P}}{\sigma_{S}}\mu_{S}.$$

The optimal treatment price therefore measures the difference between the central mass (the means) of the private and societal distributions, adjusting with the ratio of the standard deviations of each distribution to allow for differences in how that mass is spread. Effectively, this provides a measure of the “distance” between a point on each distribution which are comparable in terms of the cumulative distributions at those points. Measured from a societal value of zero (such that *φ*(T*) = 0, and treatment is allocated to everyone returning a positive societal value), the optimal treatment price is intuitively equal to this “distance”.

The definition of payment scheme $\left\{T^{*},C_{L}^{*}\right\}$ is completed by substituting equations (15), (19), (13), to give the corresponding optimal lumpsum payment as

(20) $$C_{L}^{*}=C_{tot}-\left(\mu_{P}-\frac{\sigma_{P}}{\sigma_{S}}\mu_{S}\right)N\left(1-F_{S}\left(0\right)\right).$$

As shown in Appendix B, the corresponding maximum total welfare is defined as

(21) $$W^{*}=-C_{tot}+N\left[\left(1-F_{S}\left(0\right)\right)\mu_{S}+\sigma_{S}^{2}f_{S}\left(0\right)\right].$$

Scaled by *N*, the two terms inside the square brackets here measure the societal benefit that feeds into the optimal social welfare calculation. It is evident that the probability mass of the societal value distribution at or below zero (for the first term) and at zero (for the second term) will strongly influence this calculation. At most, only one of 1 − *F*_*S*_(0) or *f*_*S*_ (0) can be close to one, and we observe that a large *f*_*S*_ (0) and a large *σ*_*S*_ are somewhat conflicting. The intuitive reasoning is therefore also most likely - that larger values of W* are associated with a societal value distribution with little probability mass at or below zero. That is, a population where only a small number of individuals would return a negative societal value from treatment.

#### 3.2.3 Optimality with imperfect dependence between societal and private values

To explore the structure of the optimal payment scheme and maximum returned social welfare in cases where there is imperfect rank correlation, we resort to numerical simulation of equation (8). We define a sample of the target population (who are candidate individuals to receive treatment) as the tuple **P** = (**P**_*P*_, **P**_*S*_). The subscripts *P* and *S* denote the private and societal values of the population, and we can express the *i*th individual of the population in terms of their private and societal values, $P_{i}=\left({P_{P}}_{i},{P_{S}}_{i}\right)$.

Following the modelling discussion in Section 2, the *i*th individual will receive treatment only if ${P_{P}}_{i}\geq T$ and the sub-populations who are allocated and refused treatment are $P^{A}\left(T\right)=\left\{P_{i}\in P|{P_{P}}_{i}\geq T\right\}$ and $P^{R}\left(T\right)=\left\{P_{i}\in P|{P_{p}}_{i}<T\right\}$, respectively. The volume-based and lumpsum (subscription) payments can therefore be extracted from equation (2) for fixed manufacturer payment *C*_*tot*_. Reformulating the social planner's welfare defined in equation (8), to represent a population of discrete individuals, gives

(22) $$W=-C_{tot}+\underset{P_{i}\in P^{A}\left(T\right)}{\sum }{P_{S}}_{i}.$$

We define the level of correlation (across the target population sample) between the private and societal value for a given individual as *ρ*. Note that for modelling simplicity, rather than using a rank correlation measure, we identify *ρ* as the stricter Pearson correlation coefficient. This may increase the impact of dependency, but avoids an additional layer of uncertainty in the sampling approach. We vary the correlation *ρ* ∈ [ − 1, 1], and for a given correlation level we generate a sample of the target population, with private and societal values for each individual. Treatment allocation, and resulting social welfare and payment composition, are then determined as the treatment price *T* varies.

We consider the case where the private and societal values are each normally distributed across the target population, and write these for the *i*th individual as ${P_{P}}_{i}∼N\left(\mu_{P},\sigma_{P}\right)$ and ${P_{S}}_{i}∼N\left(\mu_{S},\sigma_{S}\right)$, respectively. We define two vectors of independent samples from the standard normal distribution: $X_{1}∼N\left(0,1\right)$ and $X_{2}∼N\left(0,1\right)$, where each element of these vectors is sampled from the standard normal distribution. We then make the following definitions for the private and societal values of the *i*th individual:

(23) $${P_{P}}_{i}=\mu_{P}+\sigma_{P}{X_{1}}_{i},$$

and

(24) $${P_{S}}_{i}=\mu_{S}+\sigma_{S}\left(\rho {X_{1}}_{i}+\left(1-\rho \right){X_{2}}_{i}\right).$$

## 4 Practical application for novel antibiotic treatment

### 4.1 Motivation

As a practical application of the modelling presented in Section 3, we consider the case of Gonorrhoea infections in England. Gonorrhoea is a common sexually transmitted infection, and Gonorrhoea levels are at their highest for at least 11 years (BBC News, 2020). Gonorrhoea has consistently developed resistance to each proposed antibiotic treatment (see for example Whittles et al. (2017); Fingerhuth et al. (2016) and references therein). The threat posed by having no effective antimicrobial treatment for Gonorrhoea has been publicised internationally by health organisations (World Health Organization, 2012; European Centre for Disease Prevention and Control, 2019b; Centers for Disease Control and Prevention, 2012). In the UK, Public Health England has established GRASP (Gonococcal resistance to antimicrobials surveillance programme (Public Health England, 2019)) in response to this threat. A dual antimicrobial therapy of ceftriaxone and azithromycin is the only remaining recommended first-line treatment therapy; however, there have been several recent reports of antimicrobial resistance to this final robust treatment option (Bignell and Unemo, 2013; European Centre for Disease Prevention and Control, 2019b,European Centre for Disease Prevention and Control, 2019a). At present, there are no new classes of antibiotics to be deployed when this resistance becomes widespread (Davies et al., 2013).

### 4.2 Parameterising the model

To parameterise the models, we use indicative data for the case of Gonorrhoea infections in England, noting that for several of these parameters alternative values are reported in different sources. This modelling is not intended to provide specific decision-making support for the financing and deployment of a particular Gonorrhoea treatment. Rather, to use the example of Gonorrhoea treatment to elucidate the modelling presented in Section 3 and to investigate the impact on the model outputs, under alternative realisations of the societal value distribution, and varying relationships between the private and societal value distributions. A summary of the model parameter values used in the analysis is presented in Table 1, and further details on the modelling and literature sources to derive these values are provided in Appendix C. In Appendix D and Appendix E, the analysis is also extended to consider additional alternative realisations of both the private and societal value distributions. This therefore enables the analysis presented here to be translated to a variety of alternative infections and treatment contexts.

**Table 1:** Indicative values based on Gonorrhoea infection used to parameterise the model for analysis. Further details and derivation of these values is provided in Appendix C. The final column indicates the parameter values used in each subplot of Fig. 1, Fig. 2, Fig. 3. Cross-reference with Appendix C for additional parameter values.

Model input parameter	Notation	Indicative Value	Relevant subplots
Total financial outlay	*C*_*tot*_	*£*10 M	Both
Population size	*N*	70,936	Both
Mean total private value	*μ*_*P*_	*£*916.48	Both
Standard deviation of total private value	*σ*_*P*_	*£*275	Both
Mean total societal value	*μ*_*S*_	*£*1375	(i)
		*£*0	(ii)
Standard deviation of total societal value	*σ*_*S*_	*£*92	Both

### 4.3 Analysis

#### 4.3.1 Normally distributed societal and private value distributions with rank-preserving stochastic dependence

**Fig. 1:**
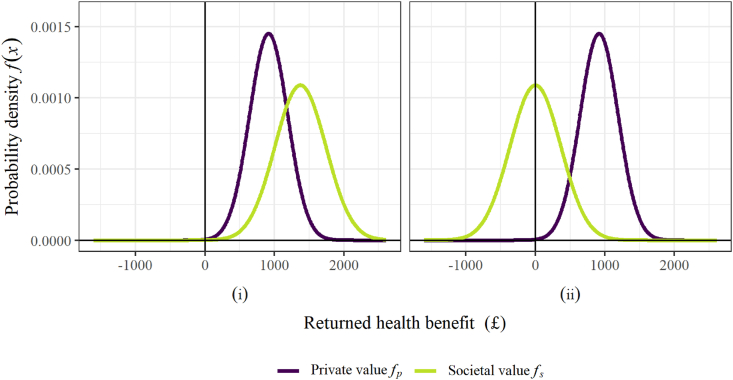
Visualisation of the private and societal value distributions, as the standard deviation of the private value and the mean societal value are varied. All parameter values are given in Table 1.

**Fig. 2:**
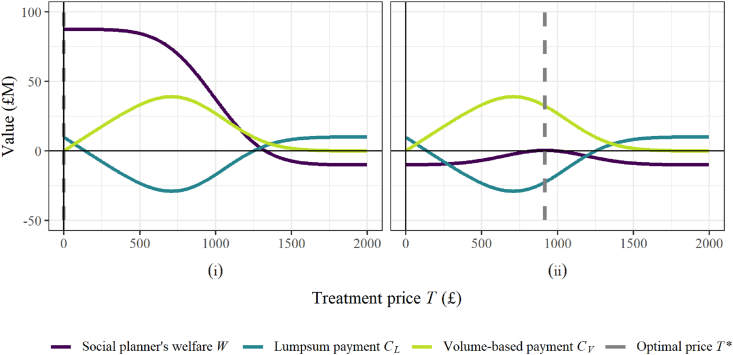
Visualisation of the optimal treatment price, the corresponding payment split between lumpsum and volume-based components, and the resulting social welfare, as the private and societal value distributions are varied via the standard deviation of the private value and the mean societal value. All parameter values are given in Table 1.

The model outlined in Section 3.2.2 is parameterised with the input values defined in Table 1. These parameter combinations specify two alternative scenarios, which are investigated to illustrate some key features of the analysis. Fig. 1 compares the private and societal value distributions for each of the scenarios, and Fig. 2 displays the key model outputs for each scenario. Fig. 1 highlights the difference between the two scenarios: in Scenario (i) (Fig. 1(i)) the central mass of the societal value distribution covers larger values than the private value distribution, whereas in Scenario (ii) (Fig. 1(ii)), the central mass of the societal value distribution is reduced, and negative values are commonly observed. A practical example of Scenario (i) is the case where the benefits from reduced transmission and increased diversity outweigh the costs of resistance to ABX emerging and growing. In contrast, for Scenario (ii) preserving ABX - that is, conserving the effectiveness of the drug by using it more sparingly - is given higher priority. In this scenario, the cost of ABX-resistant strains emerging and spreading is more prominent in the value function. Thus, for some patients, the risk that treating with ABX causes further spread of resistance to the drug outweighs the expected transmission and diversity gains.

**Fig. 3:**
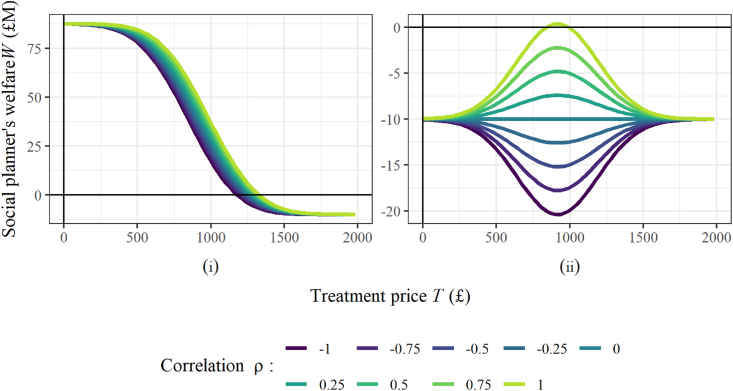
Visualisation of the impact on the social planner's welfare as the level of correlation between private and societal value distributions is varied, and the private and societal value distributions are varied via the standard deviation of the private value and the mean societal value. All parameter values are given in Table 1. For each investigations the distributions are sampled 1,000,000 times.

When large societal values are likely to be observed for most individuals, as in Scenario (i), the entire population should be treated (Fig. 2(i)). Setting the treatment price at zero would ensure that all private values are greater than this, and that treatment is allocated to the whole population. This is confirmed in Fig. 2(i), with the social welfare at its maximum at zero and the grey dashed line confirming the optimal treatment price at T* = 0. Increasing the treatment price would withhold treatment from some individuals with positive societal value and therefore reduce the total social welfare that is returned.

With preserving the antibiotic a greater concern in Scenario (ii), treatment yields smaller and potentially negative societal values, and treating the whole population can be expected to be less effective. Appendix D demonstrates that a treatment threshold at zero (treating the whole population and equivalent to a pure lumpsum (subscription) payment), would be close to optimal for cases where there is sufficient probability mass over large values of the societal value distribution that the returned health benefits exceed the manufacturers payment. As the societal value distribution shifts to the left, however, so that smaller societal values are more likely to be observed, the benefits from treating the whole population reduce. From Fig. 1(ii) the societal value distribution is centred at zero, and positive and negative societal values would therefore cancel out. As a result, Fig. 2(ii) demonstrates that at *T* = 0 the welfare is equivalent to the manufacturer payment (with no benefits gained from the treatment). A more sensible approach for Scenario (ii) is to restrict treatment to those individuals who would most benefit, and Fig. 2(ii) confirms that as the treatment is increased from zero, treatment is withheld from those that return the lowest societal values, and the social welfare increases. With the Scenario (ii) parameterisation, the optimal outcome is to only treat the individuals with the top 50% of societal values, and the grey dashed line shows the optimal treatment price is equal to the mean private value, *μ*_*P*_.

Appendix D investigates scenarios in which the societal value shifts further to the left - for example, as the costs of resistance to ABX increase further, and the requirement to conserve the effectiveness of ABX becomes more intense. The optimal approach is to treat fewer individuals, also returning lower societal values, and the maximum welfare that can be returned decreases further. The extreme case is that the need to conserve the drug outweighs any benefits from treatment, and treatment is withheld from all individuals.

From equations (12), (13), the payment components are independent from the societal value, and are therefore equivalent between Fig. 2 (i) and (ii). It is interesting to note that at the higher optimal treatment price of Scenario (ii), the volume-based payment would exceed the total payment to the manufacturer *C*_*tot*_ and would therefore incur a negative lumpsum payment - that is, from the manufacturer to the health-care funder. In practice, an alternative payment schedule may be realised instead. It is worth noting also that a practical implementation may be subscription-only from the manufacturer's perspective (with treatment price equal to zero), but that the healthcare funder may impose a treatment price on the healthcare provider, to encourage more selective allocation of treatment. Appendix D provides further detailed comparison of the social planner's welfare and the payment schedules under additional scenarios.

#### 4.3.2 Normally distributed societal and private value distributions with varying stochastic dependence

We now consider the impact on the model outputs when the stochastic dependence between the private and societal values is imperfect - in practical terms, those individuals who would most benefit from treatment are different to those individuals whose treatment would be most beneficial to society. Of the factors discussed in Appendix C which may contribute to societal value, the relationship between private value and transmission of the infection is most intuitive. An individual who would greatly benefit from treatment could be expected to present very severe symptoms. For hospital-acquired infections, the most seriously affected individuals (highest private values) could also be expected to be those individuals who present the highest societal risk of transmitting infections to other individuals with a high-risk of suffering severe consequences. Examples of this would include infections such as Carbapenem-resistant Enterobacteriaceae (CRE) and Methicillin-resistant Staphylococcus aureus (MRSA), which is also commonly acquired in community settings. In a community setting, however, exposure to higher-risk individuals could be expected to be less than in a hospital setting. Transmission would be influenced to a large extent by the behaviour of the infected individual, and their interaction with other members of the community. For example, individuals infected by sharing contaminated needles for recreational drug use may ignore their own transmission risk when suffering severe symptoms (high private values). In contrast, individuals infected by skin-to-skin contact in a sports setting may reduce their activity which would reduce their own transmission risk. The costs related to transmission risk could therefore vary greatly throughout the infected population. For sexually transmitted infections such as Gonorrhoea, it could be expected that individuals with severe symptoms would be less likely to transmit their infection to sexual partners. Instead, asymptomatic individuals (low private value) could be expected to represent the highest risk of transmission. Based purely on transmission dynamics, different infections can therefore be expected to exhibit distinct dependency relationships between the private and societal values of infected individuals, and correlation levels could be expected to vary substantially between infections. Levels of correlation closer to −1 may therefore be most realistic for Gonorrhoea treatment, and exploring Gonorrhoea treatment across the range of possible levels of dependency demonstrates the potential impact to a decision maker for other types of infection.

The simulation approach set out in Section 3.2.3 is implemented to the illustrative case of Gonorrhoea with parameter values set out in Table 1. 1,000,000 samples are drawn from the private value distribution as given by equation (23), and the level of correlation *ρ* is then varied across the range *ρ* ∈ [ − 1, 1] to generate distinct sets of samples for the societal values corresponding to each individual private value, according to equation (24). Each set of societal value samples therefore features a specific level of correlation with the private values, and for each set of samples the social welfare is calculated as the treatment price is varied.

The level of dependency between the societal and private values influences social welfare far more significantly when the antibiotic should be conserved (Scenario (ii)), than when it is used more widely (Scenario (i)). In Scenario (i), the welfare decreases with dependency, but the optimal welfare achieved is roughly equivalent across all levels of dependency (Fig. 3(i)). In Scenario (ii), however, the level of dependency has a much greater impact on the optimal welfare (Fig. 3(ii)).

As discussed in Section 4.3.1, with *ρ* = 1, the optimal social welfare is returned at T* = *μ*_*P*_. This treats half the population, and corresponds to treating all individuals returning positive societal values. As *ρ* decreases, the treated individuals are less likely to be associated with positive societal values, and the optimal social welfare decreases. With *ρ* = 0, the treated individuals are sampled according to the societal value distribution (shown in Fig. 1(ii)). In this case, any portion of the population that is treated is likely to comprise an equal balance between positive and negative societal values. With *ρ* < 0, any treatment strategy with *T* > 0 is likely to withhold treatment from some individuals with positive societal value, and the optimal strategy is therefore to treat everyone.

Fig. 3 also illustrates that the effectiveness of a treatment strategy can vary substantially between different infections. Comparing between an infection with a correlation close to 1 or close to −1 (for example, possibly relevant for MRSA or Gonorrhoea, respectively), we see that a similar treatment approach would be successful in both cases, if conserving the antibiotic is less of a requirement (see Fig. 3(i)). When the need to conserve the antibiotic is more intense (see Fig. 3(ii)) there are substantial differences in the maximum level of welfare that can be achieved, and in the optimal treatment price required to achieve this welfare. Further detailed comparison of the model outputs as the correlation is varied is shown in Appendix E.

## 5 Conclusions

In this paper we have presented and analysed a simple model of a subscription-based payment schedule for antibiotics. The key conclusion from our analysis is that, to effectively roll-out a subscription-based payment schedule for a new antibiotic, it is essential to understand the societal benefits that are gained from treatment, and in particular two features of this:

● *firstly*, what is an appropriate and comprehensive approach to measure the benefits returned for individuals for whom treatment with the particular antibiotic is medically appropriate?
● *secondly*, how do the societal benefits gained from treating an individual compare with the decision criteria that are used to determine whether that treatment should be allocated?

The analysis we present in Section 4.3, and further in Appendices B and C, highlights the substantial differences in the optimal pricing approach and the social welfare that is returned, for different realisations of this societal benefit and relationship with the treatment approach. Specifically, Section 4.3 illustrates a key policy consideration regarding a “pure” subscription payment strategy, which would correspond with the entire candidate population receiving treatment. This strategy is shown to return a close-to-optimal welfare for cases where the antibiotic's prioritised role is to reduce transmission. However, in cases where there is a more pressing requirement to preserve the antibiotic to maintain and prolong the effectiveness of the treatment as a last line of defence, we observe that this would be an extremely poor strategy. For these cases, the approach outlined in Section 4.3 can be used to inform policy makers on more effective payment strategies.

One of the key take-home messages for policy makers from this analysis is that implementing a funding schedule along the lines of Towse et al. (2017) should be subject to a test of whether this will actually improve welfare. Although making such a welfare assessment is challenging, in view of the large sums of public money involved and the potentially contrasting welfare outcomes, it is in our view incumbent on decision makers to make the attempt.

Empirical estimation of model parameters in this setting is extremely challenging, and accurate assessment of these will require investment in underpinning fundamental science. Accurate estimation of the manufacturer payment *C*_*tot*_ is also important for our model. The manufacturer payment used in this analysis is aligned with the annual payment on offer under the UK government's subscription-based scheme: up to *£*10 M per year for up to 10 years in total. This *£*100 M total from the UK could reasonably be expected to fall short of the development costs for a novel antibiotic; however, a similar response from other countries would make this a much more viable offering. In their commentary, Rex and Outterson (2020) postulate that equating this *£*100 M to the UK's “fair share” of the global value of a new antibiotic would represent a total global payment of approximately *£*3.3bn. Antibiotic resistance is a global problem, and a global solution would involve all major countries adopting a variant of the UK model.

This work is positioned to elucidate the economic practicalities of a subscription-based payment schedule that incentivises antibiotic development. The modelling framework could equally be applied, however, to other medical interventions where there is a reasonable argument for distinct valuations that drive treatment allocation and measure the returned welfare from treatment.

## Credit author statement

**Euan Barlow**: Formal analysis, Investigation, Methodology, Software, Writing – original draft, Writing – review & editing. **Alec Morton**: Conceptualization, Formal analysis, Investigation, Methodology, Funding acquisition, Writing – original draft, Writing – review & editing. **Itamar Megiddo**: Funding acquisition, Investigation, Methodology, Writing – original draft, Writing – review & editing. **Abigail Colson**: Funding acquisition, Investigation, Methodology, Writing – original draft, Writing – review & editing.
