# Supplementary material for: Optimal subscription models to pay for antibiotics
Source: Soc Sci Med. 2022 Apr;298:114818. doi: 10.1016/j.socscimed.2022.114818 (PMC9005781; doi:10.1016/j.socscimed.2022.114818)
Supplement: Multimedia component 1 [file mmc1.pdf]

Optimal subscription models to pay for antibiotics – Appendices

Appendices

A Proofs for proposition 1 and proposition 2

Proposition 1

Proof. From equation (10), $W(T)$ has a turning point at $NTf_S(T) = 0$. By definition $f_S(T) : \mathbb{R} \to [0, 1]$, and for a non-trivial problem context we can assume that $N > 0$. The turning points of W will therefore exist at $f_S(T) = 0$ and $T = 0$. From the domain of f_S, if we assume that the function has finite support then $f_S(T) \to 0$ as $T \to \pm\infty$. For $T \to -\infty$, the integrand in equation (9) tends to the expectation of f_S, and $W(T) \to C_{tot} + N\mathbb{E}[f_S]$. For $T \to \infty$, the integrand in equation (9) tends to zero as the integral range diminishes, and $W(T) \to -C_{tot}$. From equation (10), $d^2W/dt^2 = -Nf_S(T) - NTdf_S/dT$. For the domain of f_S, at $T = 0$ this is less than zero, and will therefore be a local maximum. Inspecting the integral from equation (9), $\int_{-\infty}^{\infty} xf_S(x)dx <= \int_0^{\infty} xf_S(x)dx$ for $x \in \mathbb{R}$, and the maximum welfare at $T = 0$ is therefore a global maximum. From equation (7) we define this as $T^* = 0$, and from equation (4), it follows that at T^*, $C_{tot} = C_L$. ■

Proposition 2

Proof.

1. The proof is as given for Proposition 1, with some minor adjustments. We use equation (15) to impose a perfect rank correlation between the private and societal value distributions throughout the range of T. From equation (17), $W(T)$ has a turning point at $N\phi(T)f_S(\phi(T))d\phi/dT = 0$. By definition $f_S(\phi(T)) : \mathbb{R} \to [0, 1]$, and for a non-trivial problem context we can assume that $N > 0$. The turning points of W will therefore exist at $\phi(T) = 0$, $f_S(\phi(T)) = 0$ and $d\phi/dT = 0$. From the domain of f_S, if we assume that the function has finite support then $f_S(\phi(T)) \to 0$ as $\phi(T) \to \pm\infty$. From equation (15), at its maximum $\phi(T) \to \infty$ as $\bar{F}_P(T) \to 1$, and at its minimum $\phi(T) \to -\infty$ as $\bar{F}_P(T) \to 0$. The extreme points of $\phi(T)$ (at which $d\phi/dT = 0$), therefore coincide with $f_S(\phi(T)) = 0$. For $\phi(T) \to -\infty$, the integrand in equation (16) tends to the expectation of f_S, and $W(T) \to C_{tot} + N\mathbb{E}[f_S]$. For $\phi(T) \to \infty$, the integrand in equation (16) tends to zero as the integral range diminishes, and $W(T) \to -C_{tot}$. From equation (17), $d^2W/dt^2 = -Nf_S(\phi(T))(d\phi/dT)^2$. For the defined domain, $f_S > 0$, and as $\phi(T)$ is an increasing function with positive gradient between its endpoints, at $\phi(T) = 0$ this is less than zero, and will therefore be a local maximum. Inspecting the integral from

equation (16), $\int_{-\infty}^{\infty} x f_S(x)dx <= \int_0^{\infty} x f_S(x)dx$ for $x \in \mathbb{R}$, and the maximum welfare at $\phi(T) = 0$ is therefore a global maximum. From equation (7) and equation (15) we have that $\phi(T^*) = 0 = \bar{F_S}^{-1}\bar{F}_P(T^*)$, or equivalently $T^* = \bar{F}_P^{-1}\bar{F}_S(0)$.

2. Consider an alternative function $\phi(T)$ which maps private values to societal values, but does not preserve a perfect rank correlation. Define the set of private values as $\{X_{Pi}\}$ for $i = 1, 2, \ldots, N$, and an ordering such that $X_{P1} \geq X_{P2} \geq \ldots \geq X_{PN}$. Similarly, define the set of societal values as $\{X_{Sj}\}$ for $j = 1, 2, \ldots, N$, and an ordering such that $X_{S1} \geq X_{S2} \geq \ldots \geq X_{SN}$. With an imperfect rank correlation, there exists at least one individual for which $X_{Sj} = \phi(X_{Pi})$ is such that $i \neq j$. This would require that the function $\hat{\phi}$ is not a 1-2-1 mapping, and that the gradient of $\hat{\phi}$ is not necessarily positive between the endpoints of the function range. At $\hat{\phi}(T) = 0$, the second derivative of the welfare that is shown in Proof 2.1 is therefore not necessarily negative, and the returned welfare is not guaranteed to be a maximum. Furthermore, the range of private values in the treated population, $X_P \in [T, \infty)$ is not guaranteed to map to a continuous range $X_S \in [\phi(T), \infty)$, and the integrand in equation (7) cannot necessarily be fully evaluated.

3. This is a simple consequence of stochastic dominance. It follows from the definition of stochastic dominance that $\bar{F}_P(T) \leq \bar{F}_S(T)$. Since $\bar{F}_S^{-1}$ is an increasing function, $\phi(T) = \bar{F}_S^{-1}\bar{F}_P(T) \leq T$.

4. It is a simple consequence of the hypothesis that $\bar{F}_P(T) = \bar{F}_S(T - c)$ and hence $\phi(T) = \bar{F}_S^{-1}\bar{F}_P(T) = T - c$.

■

B Deriving optimal welfare and payment scheme expressions for normally distributed value functions

In Section 3.2.2 we consider the case where the societal and private values are normally distributed as $X_S \sim \mathcal{N}(\mu_S, \sigma_S)$ and $X_P \sim \mathcal{N}(\mu_P, \sigma_P)$. Substituting standard definitions into equations (15), (16), and (17) gives, respectively

$$\phi(T) = \mu_S + \frac{\sigma_S}{\sigma_P}(T - \mu_P), \tag{1}$$

$$W(T) = -C_{tot} + \frac{N}{\sqrt{2\pi}\sigma_S}\left\{\frac{1}{2}\exp\left[-\left(\frac{T-\mu_P}{\sqrt{2}\sigma_P}\right)^2\right] + \mu_S\left[\frac{1}{2}\left(1 - \text{erf}\left(\frac{T-\mu_P}{\sqrt{2}\sigma_P}\right)\right)\right]\right\}, \tag{2}$$

and

$$\frac{\partial W}{\partial T} = -N\phi(T) f_P(T), \tag{3}$$

where erf represents the error function. The total welfare is maximised with respect to the treatment price when this derivative is zero. The optimal treatment price T^* (for non-trivial extrema of T) must therefore satisfy $\phi(T^*) = 0$, as per Proposition 2.1.

To formally define the optimal payment scheme, setting $\phi(T^*) = 0$ in equation (1) returns the optimal treatment price

$$T^* = \mu_P - \frac{\sigma_P}{\sigma_S}\mu_S, \tag{4}$$

as given in equation (19). As the societal value is normally distributed, it is convenient to exploit equation (18) to retrieve the corresponding maximum total welfare. The expectation calculation for the truncated and normally distributed societal value is defined as

$$E[X_S|X_S > 0] = \mu_S + \frac{\sigma_S^2 f_S(0)}{1 - F_S(0)},$$

and substituting this into equation (18) gives

$$W^* = -C_{tot} + N\left[(1 - F_S(0))\mu_S + \sigma_S^2 f_S(0)\right], \tag{5}$$

as given in equation (21).

C Details of model parameterisation

Table 1 presents the data that has been extracted from the literature to populate the model parameters and their sources. Further details on how these values have been processed are presented in Appendices C.1 - C.2.

C.1 Input values for the private value and context parameters

The total financial outlay is taken to be $C_{tot} = £10$M, in line with the recently publicised UK payment model (National Institute for Health and Care Excellence 2020) (see Section 5 for further discussion of this). The population size is set to $N = 70,936$, as given in (Public Health England 2019) for the total recorded number of new Gonorrhoea diagnoses in England in 2019.

The health impact of Gonorrhoea can been assessed in terms of chronic pelvic inflammatory disease (PID), which is one of the most serious complications from an infection. In Aledort et al.

Table 1: Reference data used to determine the indicative values for the model input parameters. Further details and descriptions of data use to construct the model input parameter values are provided in the text. Sources: [1]National Institute for Health and Care Excellence (2020); [2]Public Health England (2019); [3]Nherera & Jacklin (2009), and [3*] is extended from data presented in Nherera & Jacklin (2009); [4]Aledort et al. (2005); [5]Fingerhuth et al. (2016); [6]National Institute for Health and Care Excellence (2013); and [7]Whittles et al. (2017).

Input Data	Value [Source]
Total financial outlay (C_{tot})	£10M [1]
Population size (N)	70,936 [2]
Treatment costs of infection	£53.90 [3*]
Probability of developing complication	0.18 [4]
Average infection duration	0.25 years [5]
Monetised health state per QALY	£$20,000$ [6]
Average health quality loss from infection complications	0.4 QALYs [3]
Treatment costs for PID	£$2,846$ [3]
Rates of new infections per case per year	0.24 - 9.15 [5,7]

(2005), the average health state quality weight for chronic pelvic pain is given as 0.6. We convert this as a loss of 0.4 quality-adjusted life years (QALYs), as in Nherera & Jacklin (2009). The duration of a Gonorrhoea infection is calculated to be an average of 0.25 years in (Fingerhuth et al. 2016), and we assume that antimicrobial treatment of the Gonorrhoea bacterium also treats PID. To translate the health benefits into costs, we use the funding threshold of cost-effective treatment, which is set at £$20,000$ per QALY in the UK (National Institute for Health and Care Excellence 2013). We therefore calculate the monetised health benefits of avoiding chronic PID as £$0.4 \times 0.25 \times 20,000 =$ £$2,000$ per year. The treatment costs for PID are given in (Nherera & Jacklin 2009) as £$2,846$. The probability of developing chronic pelvic pain from a Gonorrhoea infection is given as 0.18 in (Aledort et al. 2005), and the expected cost of chronic PID per new Gonorrhoea case is therefore £$0.18 \times (2,000 + 2,846) =$ £872.28.

Nherera & Jacklin (2009) calculate the standard treatment costs for Gonorrhoea, comprising a consultation plus a monotherapy antibiotic treatment. To account for the dual antimicrobial therapy that is currently the front-line treatment, we extend the calculations in (Nherera & Jacklin 2009) to give a treatment cost of £53.90. The total expected cost per individual Gonorrhoea infection is therefore £916.48.

For our modelling purposes we seek to define the normally distributed private value function, and we interpret this expectation as the mean private value per individual such that $\mu_P =$ £916.48. In the analysis below we investigate standard deviations of $\sigma_P = 0.1\mu_P, 0.2\mu_P, 0.3\mu_P$, in order to explore the impact of increasing uncertainty for the private value on the welfare returned and the optimal treatment price. For illustration, in Section 4 we set $\sigma_P = 0.3\mu_P$, and explore the alternative values in Sections D and E below.

C.2 Input values for the societal value

In order to parameterise the distribution of the societal value function, we consider the structure of such a function in terms of the costs and benefits that would impact a societal value. The average societal value can be expressed as a scaling of the average private value, and we outline below how the main components of the societal value could be modelled in order to facilitate this scaling.

A key benefit is reduced transmission to future cases by treating an infected individual. We make a simple assumption that the transmission benefit is a measure of the number of future infections of Gonorrhoea which would result from an untreated case. We denote this parameter as I, and further assume that the societal cost from propagating I infections can be measured in terms of the private value per individual. We can then estimate the mean transmission benefit, per treated case, as $I\mu_P$.

A key cost to society from treating an infected individual is that this creates an opportunity for resistance to the new antimicrobial to develop. Resistance will inevitably grow through time, and the rate of growth will increase as exposure to resistant strains of the bacteria increase. Mathematically the number of cases with a resistant strain of the bacterial will be a portion of the total number of future infections, $\alpha_R I$, for $0 \leq \alpha_R \leq 1$ and α_R growing through time. The resistance cost will therefore also increase through time, proportional to $\alpha_R I$. Monetising that cost is challenging, however, as it requires pricing the value of preserving non-resistant antibiotic treatment. This is the subject of ongoing research (see for example Megiddo et al. (2019)). For simplicity, let the resistance cost be expressed, per resistant case, as a positive scaling of the mean private value, $\beta_R\mu_P$, for $\beta_R \geq 0$. This would yield a resistance cost per treated case of $\alpha_R\beta_R I\mu_P$. As this cost is structured around growing resistance, it may be feasible that there is an additional cost component to value a zero-resistance, such that the total resistance cost is given as $(\alpha_R\beta_R + \gamma_R)I\mu_P$, for $\gamma > 0$.

Other benefits may also be gained, such as diversity benefit – reducing the growth in resistance to existing antibiotic treatments by reducing selection pressure of those resistant strains of the bacteria, through increased diversity of treatments. This could also be extended to consider the knock-on impact treatment of related diseases, such as chlamydia which is a common co-infection (Bignell et al. 2013) and in some countries has the same first-line treatment option (National Health Service 2018). In its simplest form, the benefits of this diversity can be modelled similarly to the cost of resistance, in this case representing avoided resistance. The total diversity benefit per treated case is then $\alpha_D\beta_D I\mu_P$, for $0 \leq \alpha_D \leq 1$ and $\beta_D \geq 0$. Note that $\alpha_D\beta_D$ represents the total (avoided) growth of resistance and cost of that resistance, across all

other Gonorrhoea treatments. Such a simple formulation may not be measurable in practice, but with the prevalence of the current first-line treatment therapy, basing this measurement on resistance to ceftriaxone and azithromycin may serve as a reasonable estimation. Additionally, as it assumed that resistance to current treatments already exists, a zero-resistance preserving term is omitted for simplicity.

Combining the above modelling of treatment benefit, diversity benefit and resistance cost gives an approximate formulation for the average societal value as

$$\mu_S = (1 + \alpha_D\beta_D - \alpha_R\beta_R - \gamma_R)I\mu_P. \tag{6}$$

Whittles et al. (2017) and Fingerhuth et al. (2016) each present models for transmission of Gonorrhoea, which account for the number of sexual partners per person and the rate of infection per partner. In (Whittles et al. 2017) the average rate of new infections per case per year is given as 5.2 for men who have sex with men in England. Fingerhuth et al. (2016) present more granular data, which includes data on heterosexual men and women in England, and distinguishes between low and high sexual activity. The resulting rates of new infections per case per year (which can be calculated from the partner change rate multiplied by the transmission probability per partnership, within each grouping) then range between 0.2419 - 9.147. As noted in the previous discussion, however, parameterising the resistance growth rates α_D, α_R and their associated cost parameters β_D, β_R is more challenging. In broad terms it could be expected that in early years of use for the new treatment, $\alpha_D > \alpha_R$. The nature of this relationship will vary through time, however, and will be dependent on the extent to which the new treatment is deployed. The value of preserving resistance in each case would perhaps contrast this, with most value to be gained from preserving zero resistance, and this value diminishing as the resistance becomes more widespread. A reciprocal non-linear relationship between corresponding α and β could therefore be expected. Similarly, the no-resistance preserving component γ_R could be expected to be dependent on α_R, and for $\alpha_R = 0$, very large values of γ_R could be expected. Depending on the specific parameterisations of these terms and the resulting nature of the term in brackets in equation (6), it is therefore feasible that the average societal value could take a broad range of positive and negative values. This will be particularly sensitive to the values that are placed on preventing or reducing resistance to the respective treatments.

As noted above, the purpose of this analysis is to illustrate the application of the modelling presented in Section 3. We therefore do not accurately define the parameters comprising the μ_S definition, but explore the impact as these parameters result in different values for the average societal value. From the decision-making perspective of a social planner, the important consid-

erations are the optimal treatment price, the corresponding payment split between lumpsum and volume-based components, and the resulting social welfare. In order to demonstrate the impact on these metrics over different scenarios, we investigate a range of parameterisations for the societal value distribution. From equation (18), it is evident that the social welfare is directly dependent on the nature of the societal value distribution in relation to zero. From equation (13) and Proposition 2.1, the treatment cost and payments will also be dependent on the relationship between the societal and private value distributions. Furthermore, recalling the discussion in Section 3.2.2, W^* will tend to asymptotic limits when the probability mass of the societal value becomes predominantly distributed over either large positive or large negative values. We therefore consider variations to the societal value distribution, as the non-negligible support of the distribution transitions from being predominantly ranged over negative values, to being predominantly ranged over values greater than the corresponding non-negligible support of the private value distribution. Specifically, we investigate mean societal values of $\mu_S = -0.5\mu_P, 0, 0.5\mu_P, 1.5\mu_P$ in order to explore these variations. For illustration, in Section 4 we focus on $\mu_S = 0, 1.5\mu_P$, and explore the alternative values in Sections D and E below.

Parameterising the standard deviation for the societal value is also challenging to reason through. The scaling between the average societal value and average private value could be extended to a defined scaling between these values in general, such that $S \propto P$. This seems unrealistic, however, as there would inevitably be variation in I within a population of Gonorrhoea cases. Formally defining a distribution for I, however, such that the societal value is a product $S \propto I * P$ would not yield a normal distribution for the societal value. Additionally, α_R and α_D could be expected to vary through time. Conceptually, more variation in the societal value than the private value would seem appropriate giving the nature and combination of influencing factors in equation (6). We therefore take the simple approach of investigating a standard deviation for the societal value of $\sigma_S = 0.4\mu_P$, controlling this to be larger than the standard deviation for the private value.

The full range of parameter values that are investigated are given in Table 2. Note that parameter combinations (l) and (f) correspond with parameter combinations (i) and (ii), respectively, in Section 4; cross-reference with Section 4 Table 1 for clarity.

D Detailed comparison of the social planner's welfare under dependency

Extending Section 4.3, the analysis is repeated with additional parameterisations, as defined in

Table 2: Indicative values based on Gonorrhoea infection used to parameterise the model for analysis. Further details and derivation of these values is provided in Appendix C above. The final column indicates the parameter values used in each subplot of Figures 1, 2 and 4.

Model input parameter	Notation	Indicative Value	Relevant subplots
Total financial outlay	C_{tot}	£10M	All
Population size	N	70,936	All
Mean total private value	μ_P	£916.48	All
Standard deviation of total private value	σ_P	£92	(a), (d), (g), (j)
		£183	(b), (e), (h), (k)
		£275	(c), (f), (i), (l)
Mean total societal value	μ_S	−£458	(a), (b), (c)
		£0	(d), (e), (f)
		£458	(g), (h), (i)
		£1375	(j), (k), (l)
Standard deviation of total societal value	σ_S	£92	All

Table 2, investigating 12 distinct combinations of the distribution parameters. In the scenarios shown in Figure 1, as the mean societal value increases, the benefits from reduced transmission and increased diversity increasingly dominate the costs of emergent or increasing resistance. Figure 2 demonstrates that these increases have a substantial impact on the modelling outputs. As the private value standard deviation, σ_P, increases, a larger range of private values can be expected. For treatment prices $T > \mu_P$, the same treatment price will therefore treat larger portions of the population, and the converse holds true for $T < \mu_P$. It is apparent from Figure 2 that the impact of changing σ_P has limited impact on the modelling outputs.

Recall that the optimal treatment price $T^* = \phi^{-1}(0)$ – that is, T^* is the price which would split the private value distribution into the same proportions as the y-axis splits the societal value distribution. From Figure 1 we can intuitively expect that T^* will increase moving from left to right along each row, as a higher price is required to treat the same portion of cases. Moving down each column, we can intuitively expect that T^* will decrease, as the y-axis intersects closer to the left-hand tail of the societal value distribution. Specifically, more cases become treated at optimality, and so the corresponding treatment price must reduce to facilitate more accessible treatment. These behaviours are confirmed in Figure 2.

Recalling from equations (12) - (13) that the payment components are independent of the societal value, these are consistent within each column of Figure 2. The maximum volume-based payment is made when T is sufficiently small that a large portion of the population are treated, but where each treatment is sufficiently expensive that the cumulated cost is relatively large. The impact of varying σ_P on the maximum volume-based payment will be dependent on the mean private value. As noted in Section 4.3.1 some values of the treatment price result in

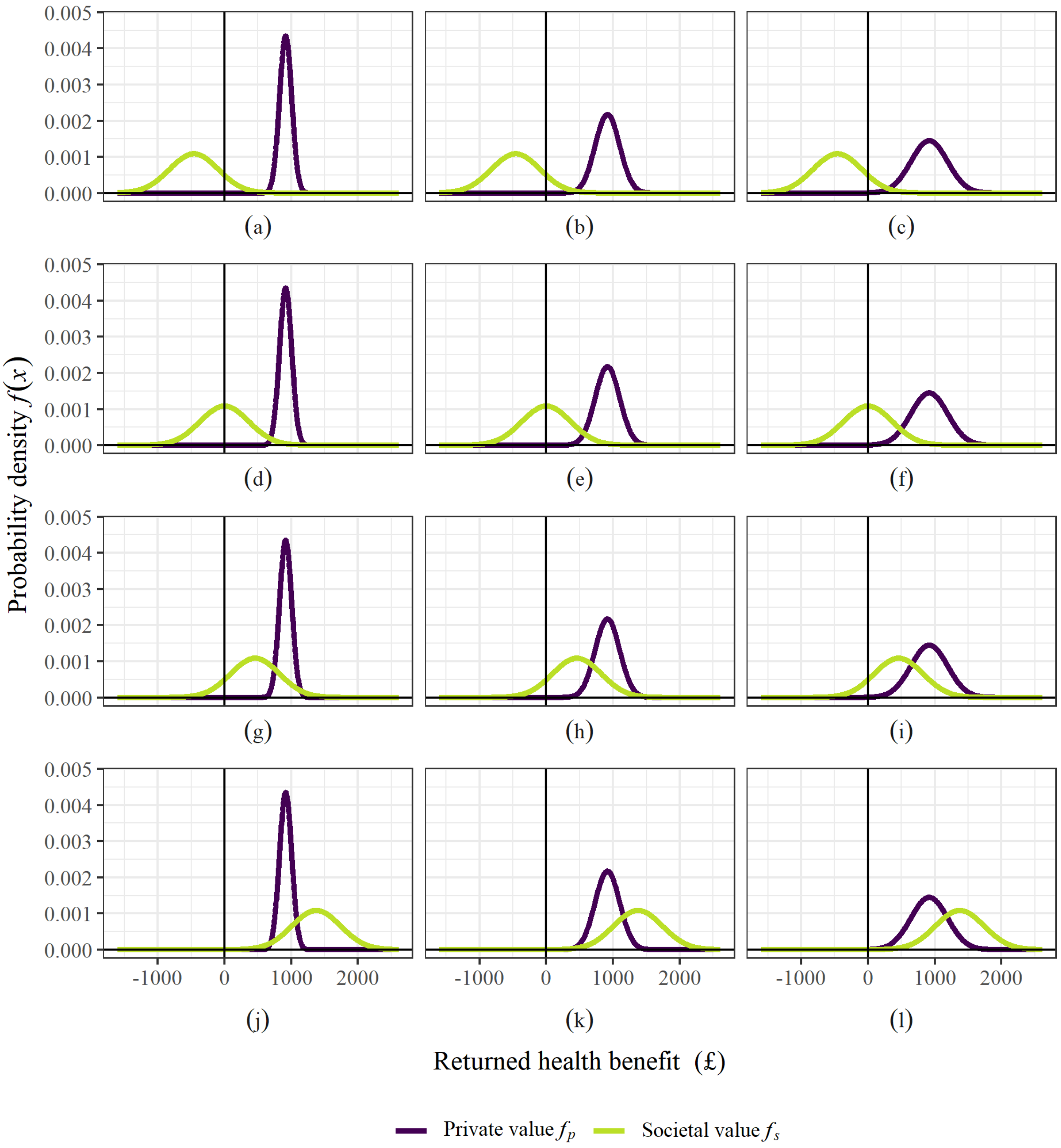

Figure 1: Visualisation of the private and societal value distributions, as the standard deviation of the private value and the mean societal value are varied. All parameter values are given in Table 2.

volume-based payments greater than C_{tot} and would therefore incur a negative payment – for Figures 2 (d)-(k) it is clear that at T^* the lumpsum payment is negative.

It is evident from Figure 2 that, for each scenario (a)-(l), there are essentially two levels of social planner's welfare, one for each extreme of T. With higher treatment prices fewer cases are treated and only small health benefits are returned, and the welfare therefore approaches the lumpsum payment of $-C_{tot} = -£10\text{M}$. At the other extreme, a treatment price close to zero will allocate treatment to the majority of cases – with private value assumed to be non-negative. When $\mu_S = 0$ (Figures 2(d)-(f)), the positive and negative societal values are equally balanced

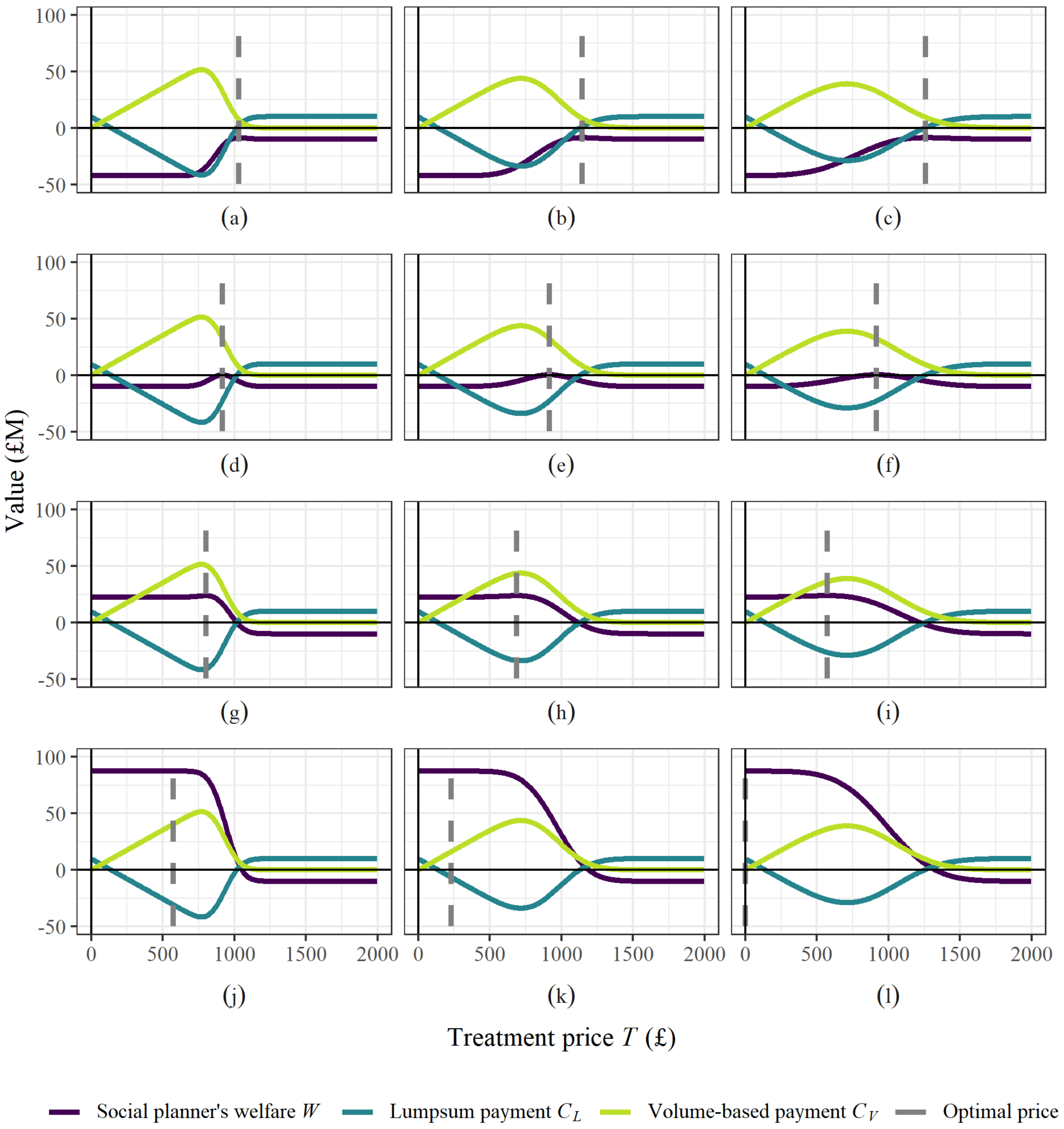

Figure 2: Visualisation of the optimal treatment price, the corresponding payment split between lumpsum and volume-based components, and the resulting social welfare, as the private and societal value distributions are varied via the standard deviation of the private value and the mean societal value. All parameter values are given in Table 2.

such that the total health benefit received is zero, and the welfare again approaches a lumpsum payment of $-C_{tot} = -£10$M. As μ_S increases (decreases) from zero, the positive (negative) societal values will dominate, and the social welfare increases (decreases).

To illustrate in more detail the behaviour of the welfare as the treatment price varies, Figure 3 focuses on the social planner's welfare for each of the defined distributions for the private and societal values discussed above. For each variant of the distributions, the welfare is normalised to the interval $[0, 1]$ to aid comparison. The discussion points above in terms of impact of changes

in σ_P and μ_S are clearly shown. In Figure 3(d) the welfare is shown to be approximately optimal for a large range of T. This is also true in Figures 3(a) and (c); however, with non-monotonic transition between the two levels of welfare for extreme values of T, setting T to the appropriate extreme value would return sub-optimal welfare. Taken in conjunction with the preceding discussion, Figure 3 highlights the importance to a social planner from ascertaining a good approximation to the societal value, as there are contrasting impacts from choosing $T^* - \delta$ and $T^* + \delta$, for relatively small $\delta \in: \mathbb{R}$.

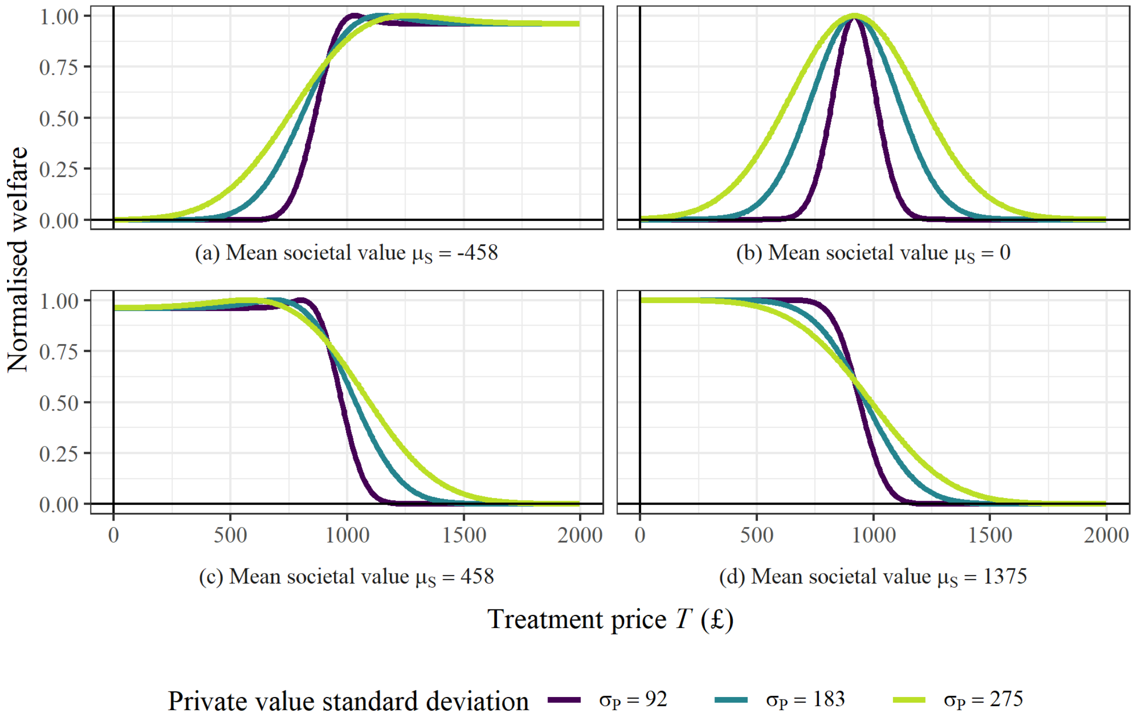

Figure 3: Visualisation of the social planner's welfare, normalised to the interval $[0, 1]$, as the private and societal value distributions are varied via the standard deviation of the private value and the mean societal value (varied as shown). All other parameter values are given in Table 1.

E Detailed comparison of model outputs with varying dependency

The simulation outputs from Section 4.3.2 are extended in Figure 4, where each of the subplots (a)-(l) corresponds to the same model parameterisations used in Appendix D. The features discussed in Section 4.3.2 are shown to be vary between the two scenarios presented there, as the parameter values vary. With independent samples of societal values and treatment price $T = 0$, increasing (decreasing) $\mu_S > 0$ ($\mu_S < 0$)), positive (negative) societal values become more prominent, and the social welfare increases (decreases). The main effects of increasing T

and varying σ_P are as discussed for Appendix D.

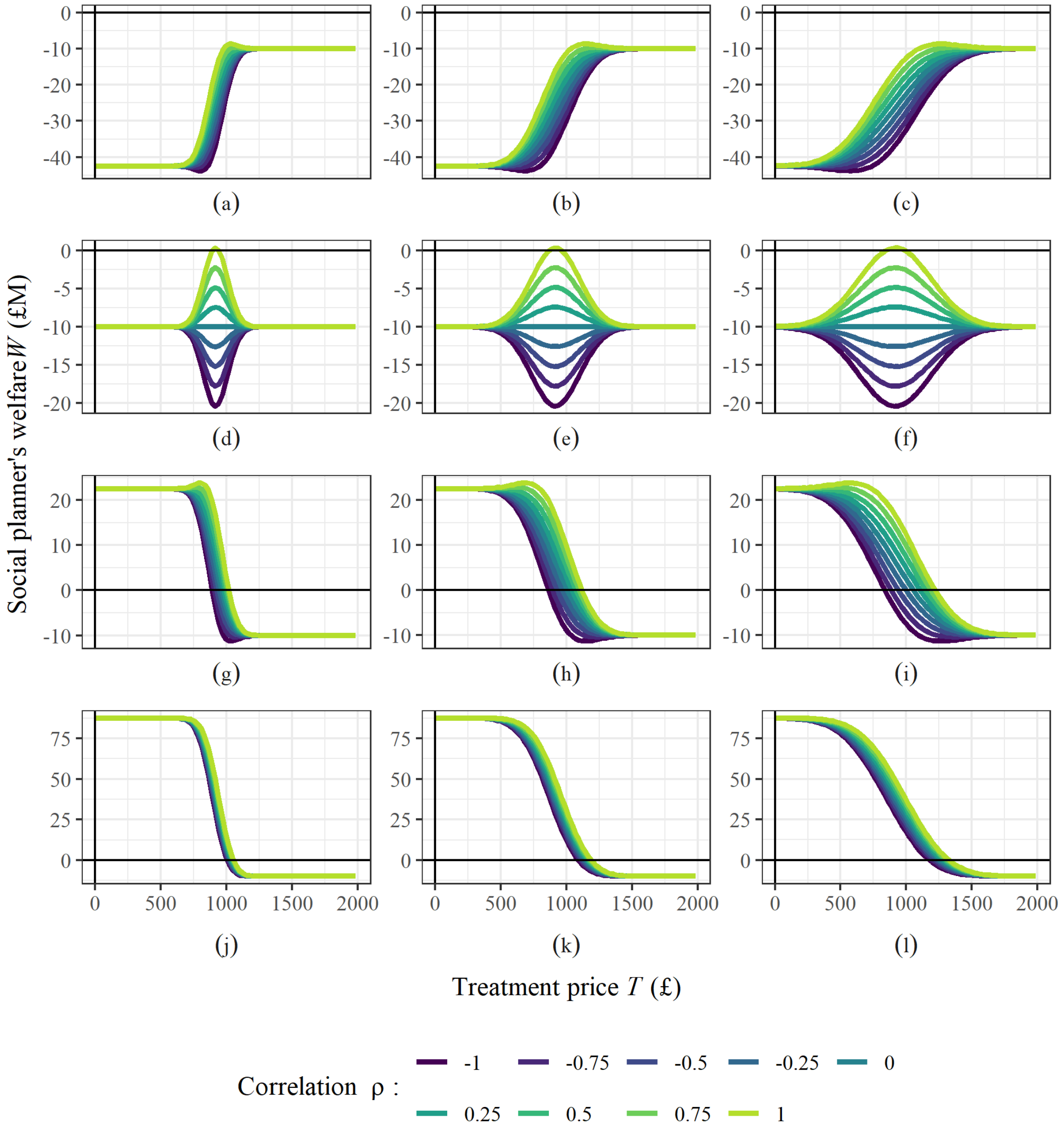

Figure 4: Visualisation of the impact on the social planner's welfare as the level of correlation between private and societal value distributions is varied, and the private and societal value distributions are varied via the standard deviation of the private value and the mean societal value. All parameter values are given in Table 2. For each investigations the distributions are sampled 1,000,000 times. Note that the y-axis scale varies between rows.

Figures 5 and 6 illustrate this behaviour further, extracting the optimal welfare value and treatment price as the level of correlation varies. As discussed for $\mu_S = 0$ (Figure 5(b)), the optimal welfare is consistently equal to the manufacturer payment for $\rho \leq 0$, and increases with ρ for $\rho > 0$. The difference between different levels of correlation is also largest for this case. The fluctuation shown in Figures 6(b) for T^* is partially a factor of the numerical simulations,

with marginal differences in private values sampled above or below μ_P translating into marginal differences in the preferred treatment strategy for ρ close to zero. For the remaining parameter investigations, the behavior of the optimal welfare is related to the behaviour of the welfare per correlation level, as the treatment price transitions between the two extreme scenarios of treating every individual or no individual.

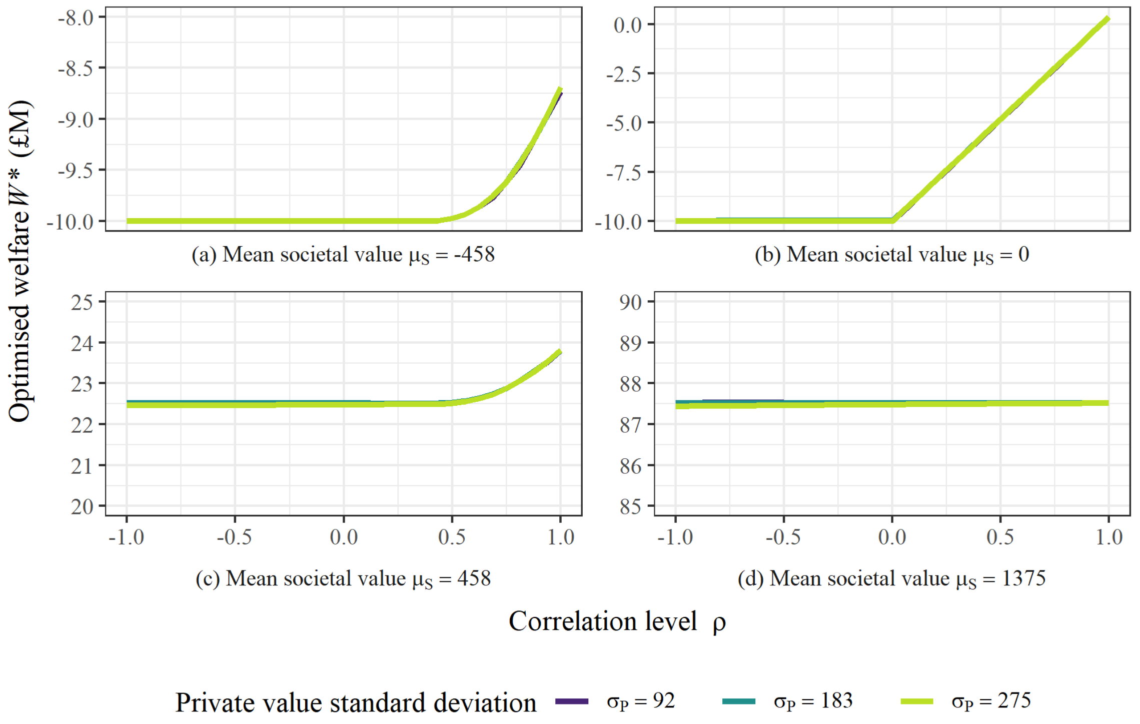

Figure 5: Visualisation of the optimised social welfare as the level of correlation between private and societal value distributions is varied. The private and societal value distributions are varied via the standard deviation of the private value and the mean societal value (varied as shown). All other parameter values are given in Table 1. Note that the y-axis scale varies between rows.

References

Aledort, J. E., Hook III, E. W., Weinstein, M. C. & Goldie, S. J. (2005), 'The cost effectiveness of gonorrhea screening in urban emergency departments', Sexually transmitted diseases **32**(7), 425–436.

Bignell, C., Unemo, M. & European STI Guidelines Editorial Board (2013), '2012 european guideline on the diagnosis and treatment of gonorrhoea in adults', International journal of STD & AIDS **24**(2), 85–92.

Fingerhuth, S. M., Bonhoeffer, S., Low, N. & Althaus, C. L. (2016), 'Antibiotic-resistant

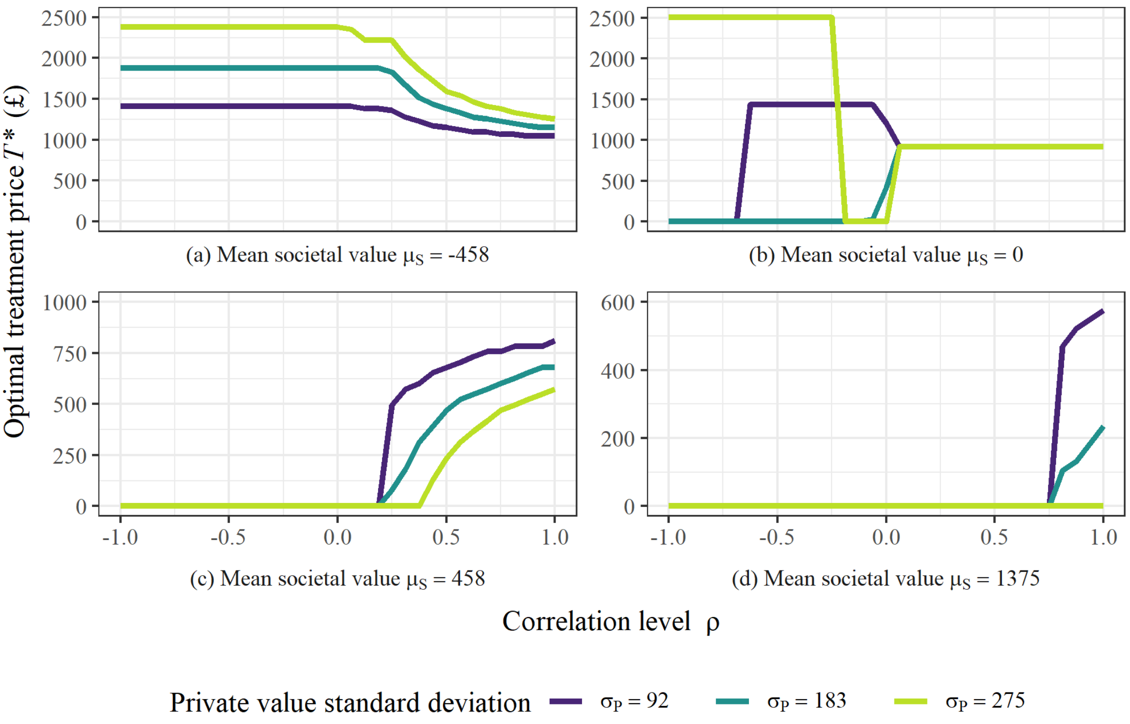

Figure 6: Visualisation of the optimal treatment price as the level of correlation between private and societal value distributions is varied. The private and societal value distributions are varied via the standard deviation of the private value and the mean societal value (varied as shown). All other parameter values are given in Table 1. Note that the y-axis scale varies between rows.

Neisseria gonorrhoeae spread faster with more treatment, not more sexual partners', PLoS pathogens **12**(5), e1005611.

Megiddo, I., Drabik, D., Bedford, T., Morton, A., Wesseler, J. & Laxminarayan, R. (2019), 'Investing in antibiotics to alleviate future catastrophic outcomes: What is the value of having an effective antibiotic to mitigate pandemic influenza?', Health economics **28**(4), 556–571.

National Health Service (2018), 'Treatment – chlamydia'. Retrieved 11/12/2020 from https://www.nhs.uk/conditions/chlamydia/treatment/.

National Institute for Health and Care Excellence (2013), 'Guide to the methods of technology appraisal 2013'. Sections 5.1.13 & 6.3. Retrieved 01/11/2020 from https://www.nice.org.uk/process/pmg9/chapter/foreword.

National Institute for Health and Care Excellence (2020), 'Developing and testing innovative models for the evaluation and purchase of antimicrobials: subscription-based payment model'. Retrieved 01/11/2020 from https://amr.solutions/wp-content/uploads/2020/03/2020-03-25-NHS-AMR-Market-Engagement-Briefing-Final.pdf.

Nherera, L. & Jacklin, P. (2009), A model to assess the cost-effectiveness of alcohol education developed for NICE public health guidance on personal, social, health and economic (PSHE) education, National Collaborating Centre for Women's and Children's Health. Retrieved 01/11/2020 from https://www.nice.org.uk/guidance/gid-phg0/documents/pshe-additional-consultation-on-the-evidence-alcohol-cost-effectiveness-modelling-report2.

Public Health England (2019), 'Official statistics; sexually transmitted infections (STIs): annual data tables; Table 1: STI diagnoses and rates in England by gender, 2010 to 2019'. Retrieved 01/11/2020 from https://www.gov.uk/government/statistics/sexually-transmitted-infections-stis-annual-data-tables.

Whittles, L. K., White, P. J. & Didelot, X. (2017), 'Estimating the fitness cost and benefit of cefixime resistance in Neisseria gonorrhoeae to inform prescription policy: a modelling study', PLoS medicine **14**(10), e1002416.
